# Effective Immune Protection of Mice from Murine Cytomegalovirus Infection by Oral *Salmonella*-Based Vaccine Expressing Viral M78 Antigen

**DOI:** 10.3390/vaccines13020137

**Published:** 2025-01-28

**Authors:** Yujun Liu, Hao Gong, Jiaming Zhu, Fenyong Liu

**Affiliations:** 1 School of Public Health, University of California, Berkeley, CA 94720, USA; 2 Program in Comparative Biochemistry, University of California, Berkeley, CA 94720, USA

**Keywords:** congenital infections, cytomegalovirus, gene delivery, gene therapy, oral vaccine, *Salmonella*

## Abstract

**Background**: Human cytomegalovirus (CMV) is the most common cause of viral congenital infections worldwide. The development of effective vaccines against human CMV infection and disease is a high priority. Attenuated *Salmonella* are attractive oral vaccine vectors against human diseases because they can be administrated orally. **Methods**: In this study, an attenuated *Salmonella* strain was generated as an oral vaccine vector for the delivery and expression of the M78 protein of murine cytomegalovirus (MCMV). Using the MCMV infection of mice as the CMV infection model, we characterized the immune responses and protection induced by the constructed *Salmonella*-based vaccine. **Results**: The generated *Salmonella*-based vaccine, v-M78, which contained an M78 expression plasmid construct, carried out gene transfer efficiently for M78 expression and showed little pathogenicity and virulence in mice. In orally vaccinated mice, v-M78 induced anti-MCMV serum IgG and mucosal IgA responses and also elicited anti-MCMV T cell responses. Furthermore, mice immunized with v-M78 were protected from intraperitoneal and intranasal challenges with MCMV. The v-M78 vaccination reduced the titers of the challenged viruses in spleens, livers, lungs, and salivary glands. **Conclusions**: These results provide the first direct evidence that a *Salmonella*-based vaccine expressing M78 elicits strong humoral and cellular immune responses and induces immune protection against MCMV infection. Furthermore, our study demonstrates the potential of using *Salmonella*-based oral vaccines against CMV infection.

## 1. Introduction

Human cytomegalovirus (CMV), called HCMV, is an important human herpesvirus that causes common congenital infections in neonates [1,2] and severe complications in immunocompromised patients such as HIV-positive individuals [3,4]. HCMV affects 1 in 200 of all live-born infants in high-income countries and 1 in 71 in low- and middle-income countries [5,6]. The development of effective vaccines against human CMV infection and disease is a high priority and significant efforts on this front are needed [7]. There is currently no FDA-approved anti-HCMV vaccine available.

HCMV is species-specific in its host range and only replicates and propagates in human cells but not in non-human cells [3]. The infections of animals by related cytomegaloviruses, such as the infection of mice by murine cytomegalovirus (MCMV) and the infection of non-human primates by rhesus CMV, serve as excellent models for studies of the immunology and pathogenesis of HCMV infection and for anti-CMV vaccine development [8]. Vaccines being explored against CMV include conventional inactivated whole-viral antigen vaccines, recombinant protein vaccines, virus-like particle (VLP) vaccines, DNA vaccines, and vaccines based on live attenuated virus vectors [7]. Using the MCMV infection of mice as the model, some constructed vaccines, when expressing different viral antigens, have elicited strong and effective immune responses against MCMV infection in mice [9,10,11,12,13]. The continued development of a novel and effective anti-CMV vaccine is crucial for the control and prevention of HCMV infection and its associated diseases.

Oral vaccines represent an excellent choice for mass vaccination because they eliminate the requirement for the use of needles and syringes. Vaccine vectors based on attenuated *Salmonella* strains have been used to deliver nucleic acid-based vaccines orally [14,15,16]. Constructed *Salmonella* mutants carry plasmid constructs containing transgenes and release the plasmid DNA in infected cells, leading to transgene expression [17,18]. Further studies have shown that the intracellular survival and replication of *Salmonella* requires the expression of the components of the type III secretion system (T3SS) of *Salmonella* pathogenicity island 2 (SPI-2) [19,20]. The inactivation of these genes increases bacterial lysis and improves gene transfer [17,21].

Here, we report the construction of a new attenuated *Salmonella* strain, SL416, with a deletion in the ssrA/B gene, which plays a key role in regulating the expression of many SPI-2 genes [22]. We generated a functional SL416-based vaccine, v-M78, that expressed the MCMV M78 protein. M78 and its HCMV counterpart, UL78, encode G protein-coupled receptor (GCR) homologs that are found in both virions and infected cells [23]. Previous studies have suggested the role of M78 in viral gene expression and pathogenesis. M78 functions to facilitate viral IE mRNA expression and down-regulates MHC II expression, while viral mutants inactivating M78 are attenuated in viral production in macrophages and defective in MCMV colonization in the salivary gland in mice [24,25]. Because they are virion proteins, M78 and UL78 are considered potential vaccine candidates against CMV infection. However, it has not been reported whether M78 or UL78 can serve as an antigen for vaccine development.

In this study, we show that the generated *Salmonella*-based vaccine, v-M78, which contained an M78 expression construct, carried out gene transfer efficiently for M78 expression and demonstrated little ability to kill mice in vivo. In orally vaccinated mice, v-M78 induced anti-MCMV serum IgG and mucosal IgA responses and elicited anti-MCMV T cell responses. Furthermore, the mice immunized with v-M78 were protected from intraperitoneal and intranasal challenges with MCMV. Our study demonstrates the potential of using *Salmonella*-based oral vaccines against CMV infection.

## 2. Materials and Methods

### 2.1. Virus Strains, Antibodies, and Expression Constructs

Mouse NIH 3T3 cells and J774 macrophages and the wildtype MCMV Smith strain were obtained from the American Type Culture Collection (Manassas, VA, USA) [26,27]. The cells were maintained in Dulbecco’s modified Eagle medium (DMEM) (Thermo Fisher, Waltham, MA, USA) supplemented with 10% NuSerum (Becton Dickinson, San Jose, CA, USA). The Smith strain and mutant m-M78 were propagated in NIH 3T3 cells [26,27]. Mutant m-M78, which contained a deletion of the M78 open reading frame (coordinates of 110989–112401) [28], was constructed using the BAC-mid method as previously described [24,25,29,30]. The M78 deletion in m-M78 was confirmed through Southern blot and sequencing analyses. The polyclonal rabbit anti-M78 antibody was generated using M78 peptides (Promab, Inc., Richmond, CA, USA) and the anti-actin antibody was from Sigma (St. Louis, MO, USA) [25,31]. To generate the expression construct peM78, the M78 encoding sequence was amplified from purified MCMV (Smith) viral DNA through PCR using the primers 5M78 (5′-GGGAATTCCATATGAAGATCTCTCCGACTTCATCGTGCGCCGT-3′) and 3M78 (5′-CCGGAATTCGGTACCTCAGACAACAGAGGAGGAGGTA-3′). The PCR product was digested with restriction enzymes and cloned into the eukaryotic expression vector peVAX, which was derived from the construct pVAX1 (Invitrogen, Carlsbad, CA, USA) with additional cloning sites, to generate the expression construct peM78.

### 2.2. Salmonella Strains

Using the λ red recombinase-based approach [32], we constructed strain SL416 from *Salmonella typhimurium* aroA strain SL7207 (a gift from Bruce A. D. Stocker, Stanford University, Stanford, CA, USA) by removing a part of the SsrA/B coding sequence [33]. Using pKan-clone7 as the template, PCR was performed with the primers ssrAB5 (5′-TGTACTGCGATAGTGATCAAGTGCCAAAGATTTTGCAACAGGCAACTGGAGGGAAGCATTCATATGAATATCCTCCTTAGTTC-3′) and ssrAB3 (5′-CTGCGTGGCGTAAGGCTCATCAAAATATGACCAATGCTTAATACCATCGGACGCCCCTGGTGTGTAGGCTGGAGCTGCT T-3′). We transformed the PCR products into SL7207 and constructed the ssrA/B deletion mutant using the λ red recombinase method [32] as described previously [34,35]. We selected the nonpolar strain SL416 based on its sensitivity to kanamycin and sequencing analysis. We performed the growth of the *Salmonella typhimurium* clinical strain ST14028s and other *Salmonella* strains in LB following published procedures [34,35].

### 2.3. M78 Expression Through Salmonella-Mediated Delivery in Cultured Cells

The *Salmonella*-based vaccines v-M78 and v-C were produced by transforming SL416 with peM78 and the empty vector peVAX, respectively. We cultured J774 cells (1 × 10^6^ cells) in the presence of IFN-γ (150 U/mL) (R&D Systems Inc., Minneapolis, MN, USA) and then infected them with *Salmonella* and harvested the infected cells at 72 h postinfection. We conducted Northern and Western blot analyses as described previously [17,34]. The protein samples were separated on SDS-containing gels and reacted with anti-M78 and anti-actin antibodies and imaged with a STORM840 Phosphorimager [17,34].

### 2.4. Oral Immunization of Mice

Four-week-old male BALB/c mice (5–10 mice per group) (Jackson Laboratory, Bar Harbor, ME, USA) were immunized on days 0, 14, and 28. We anesthetized the animals with isoflurane. Using a gavage needle, we then intragastrically inoculated the animals with phosphate-buffered saline (PBS) containing no *Salmonella* or 1 × 10^9^ cfu v-M78 or v-C [34,35]. Two trials were conducted with a total of 60 animals and each trial included 30 animals (10 mice per group).

### 2.5. MCMV-Infected Cell Lysate ELISA

Sera and mucosal samples were drawn from mice at indicated time points, following the procedures described previously [36,37]. Blood was collected and transferred to microtainer tubes (Becton Dickinson, San Jose, CA, USA) based on the manufacturer’s recommendations. Nasal wash samples were collected and prepared as described previously [38]. The serum anti-MCMV IgG and IgA antibody reactivity was measured against cell lysates infected with the wildtype MCMV Smith strain or mutant m-M78. To prepare the cell lysates, NIH3T3 cells were infected with the Smith strain or mutant m-M78 (MOI = 1). After 4 days, these cells were harvested and subjected to three freeze/thaw cycles, quantified by measuring the absorbance at 280 nm in a spectrophotometer, and stored at −80 °C, following the procedures described [26,27].

We conducted ELISAs using Medisorp plates (Thermo Fisher, Waltham, MA, USA), following the manufacturer’s recommendation. The plates were first incubated with the cell lysate (50 µg/mL) and then reacted with 100 µL per well of the serum or mucosal nasal wash samples diluted in an ELISA dilution buffer (PBS containing 5% Bovine Serum Albumin). After the antiserum incubation, the plate was then incubated with goat anti-mouse IgG (H+L) AP or anti-mouse IgA AP secondary antibodies (Cell Signaling Technologies, Danvers, MA, USA), then reacted with a chemiluminescent TMB substrate (BioLegend, California, San Diego, CA, USA) and analyzed in a plate reader (Molecular Devices, San Jose, CA, USA). The assays were performed in duplicate, and the experiments were repeated three times. The values obtained were the averages from these experiments.

### 2.6. T Cell ELISPOT Assay

We employed the mouse IFN-γ ELISPOT kit (U-Cytech biosciences, Utrecht, The Netherlands) to quantify IFN-γ-expressing T cells, following the manufacturer’s recommendations [39]. The ELISPOT plates containing mouse splenocytes (*n* = 1 × 10^6^ cells) were incubated in the absence and presence of m-M78- or Smith-infected cell lysates (150 µg/well). We then treated the plates with an anti-mouse IFN-γ antibody and analyzed the plates with a video camera [39]. Phytohemagglutinin (PHA, 4 µg/mL, Sigma–Aldrich, St. Louis, MO, USA) served as the positive control. We conducted the experiments in duplicate and repeated them three times.

### 2.7. Studies of Immunized Mice Challenged with MCMV

Ten mice per group were infected intraperitoneally or intranasally with 100 µL PBS containing 1 × 10^6^ PFU of the salivary gland-passaged MCMV Smith strain (for a lethal dosage challenge) or 5 × 10^4^ PFU of the cultured cell-passaged MCMV Smith strain two weeks after the final vaccination [26,27]. Survival studies were conducted as described previously [26,27], with the animals monitored daily for 16 days.

To quantify the level of MCMV in various organs of the infected animals, we collected the spleens, livers, lungs, and salivary glands at 5 days postinfection [26,27]. We titered these virus samples in NIH 3T3 cells in the presence of fresh DMEM (Thermo Fisher, Waltham, MA, USA) containing 1% low-melting agarose (Catalog Number: A9045-5G) (Sigma–Aldrich, St. Louis, MO, USA) and counted the plaques, following the previously described procedures [26,27]. We titered each sample in duplicate and repeated them three times.

### 2.8. Statistical Analysis

All the assays were performed in duplicate and repeated three times. We analyzed the findings with the analysis of variance (ANOVA) (GraphPad Prism software, version 10). We considered a *p*-value of <0.05 as statistically significant.

## 3. Results

### 3.1. Generation of Salmonella-Based Vaccine

We previously used *Salmonella*-based vectors to express therapeutic ribozymes or RNAs in cultured cells and in mice [17,40]. In this study, we generated an attenuated *Salmonella* strain, SL416, from *Salmonella typhimurium* strain SL7207 [33] with a deletion of part of the SsrA/B coding sequence. Previous studies have shown that SL7207 is an attenuated strain with gene delivery activity for the expression of ribozymes and small therapeutic RNAs [17,40]. The ssrA/B protein regulates the expression of many SPI-2 encoded genes important for the intracellular survival and virulence of *Salmonella* in vivo [22,41]. To generate a *Salmonella* vaccine expressing the MCMV antigen M78, SL416 was transformed with the plasmid construct peM78, which contained the M78 encoding sequence under the control of a eukaryotic expression promoter. We generated two vaccines for the study. The functional vaccine v-M78 contained strain SL416 with the construct peM78. The control vaccine v-C contained SL416 with an empty vector construct without any MCMV sequences and was used as a negative control.

### 3.2. The Characterization of the Constructed Salmonella-Based Vaccines for Their Growth and Gene Delivery Ability in Cultured Cells and in Mice

**Figure 1:**
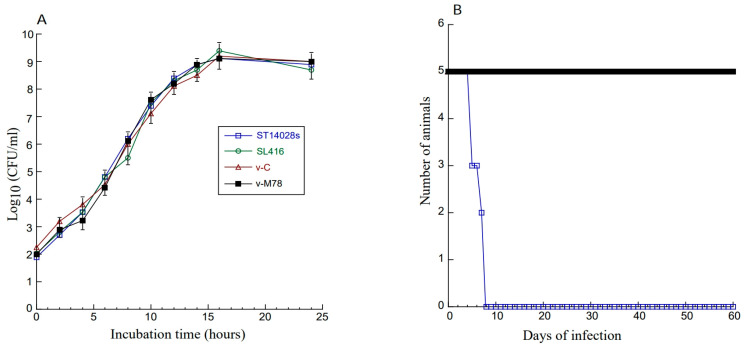
(**A**) Growth of *Salmonella* clinical strain ST14028s, mutant SL416, and SL416 with empty vector peVAX (v-C) and construct peM78 (v-M78) in LB broth. (**B**) Survival of BALB/c mice infected with *Salmonella* strains. Mice (5 animals per group) were inoculated intragastrically with ST14028 (2 × 10^3^ CFU) and SL416 (1 × 10^9^ CFU) without any construct or with empty vector and construct peM78. Experimental details can be found in Materials and Methods.

Three series of experiments were performed to characterize the constructed *Salmonella*-based vaccines. First, in vitro experiments showed that v-M78 and v-C grew in LB broth as well as the *Salmonella* clinical strains ST14028s and SL416 without any constructs (Figure 1A). Thus, the M78 sequence and vector construct did not impair the viability of the bacterial carrier.

Second, we examined the virulence of these constructed *Salmonella*-based vaccines in mice in vivo. Our results showed that SL416 carrying no construct, v-C, and v-M78 showed little virulence in killing mice compared to the clinical strain ST14028s. No death was recorded for mice infected with SL416, v-M78, and v-C (1 × 10^9^ cfu/mouse) even at 60 days postinoculation (Figure 1B). On the contrary, mice with a lower dose of ST14028s (2 × 10^3^ cfu/mouse) died within 7 days (Figure 1B). Thus, the constructed SL416-based vaccines were severely attenuated in mice and showed little ability to kill the mice.

Third, we determined if v-M78 exhibited an efficient gene delivery ability for M78 expression in cultured cells and in mice. In Northern blot analyses, neither the M78 RNA transcript nor the M78 protein was detected in v-M78 when grown in LB in vitro, indicating that M78, which was under the control of a eukaryotic expression cassette, was not expressed when the SL416-based vaccine grew outside of mammalian cells.

**Figure 2:**
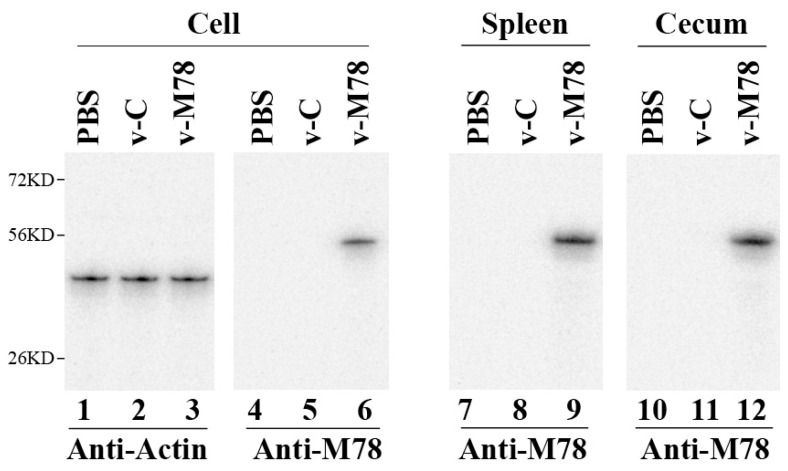
Western blot detection of MCMV M78 in cells and mice treated with phosphate-buffered saline (PBS) only (lanes 1, 4, 7, and 10), v-C (lane 2, 5, 8, and 11), and v-M78 (lanes 3, 6, 9, and 12). Cells were collected at 72 h post treatment, while spleens and cecums were isolated from animals intragastrically inoculated with PBS, v-C, or v-M78 4 days after inoculation. Protein samples (30 μg) were separated on denaturing gels and stained with anti-actin (lanes 1–3) and anti-M78 antibodies (lanes 4–12).

To determine whether v-M78 could deliver the M78 sequence into mammalian cells, mouse J774 macrophages were infected with v-M78 and v-C. At 72 h postinfection, the M78 protein expression was assayed in Western blot experiments using actin as the loading control (Figure 2). The control vaccine, v-C, in which SL416 carried an empty vector, showed no M78 expression (Figure 2, lanes 5). The M78 (~52 KD) protein was detected in cells infected with the functional *Salmonella*-based vaccine, v-M78, in which SL416 carried the construct peM78 (lane 6).

To investigate the SL416-mediated gene delivery for M78 expression in vivo, we intragastrically inoculated BALB/c mice with the vaccines v-M78 and v-C. We detected the M78 protein in the spleens and cecums of the v-M78-treated mice (Figure 2, lanes 7–12). In sum, these results indicated that v-M78 carried out gene transfer for M78 expression and showed little virulence and pathogenicity in mice.

### 3.3. Humoral Responses Elicited by Salmonella-Based Vaccines

The animals were either intragastrically treated with phosphate-buffered saline (PBS) (as the negative controls) or vaccinated with v-M78 and v-C at days 0, 14, and 28. Two trials were conducted with a total of 60 animals, and each trial included 30 animals (10 mice per group). No animal death was recorded at day 42 post immunization (i.e., two weeks after the final immunization), consistent with our results (Figure 1B) showing that v-M78 and v-C exhibited little capability to kill mice in vivo.

**Figure 3:**
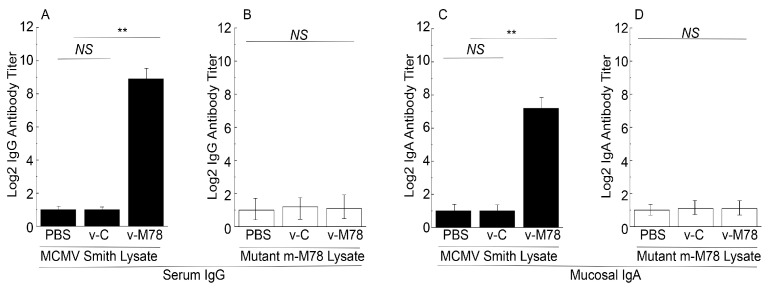
Humoral responses in mice treated with PBS, control vaccine v-C, and functional vaccine v-M78. We used ELISA to assay titers of serum IgG against lysates of cells infected with MCMV Smith (**A**) and mutant m-M78 (**B**) and titers of mucosal IgA against lysates of cells infected with MCMV Smith (**C**) and mutant m-M78 (**D**) in immunized mice at 42 days post immunization. We treated animals intragastrically with PBS only, v-C, and v-M78 at days 0, 14, and 28. Pooled serum or mucosal wash samples from mice were analyzed. ** *p* < 0.05. NS, not significant. Error bars indicate standard deviation. Two trials were conducted with total of 60 animals, and each trial included 30 animals (10 mice per group). Experiments were conducted in duplicate and repeated three times.

We performed an ELISA to assess the levels of serum antibodies at 0, 16, 32, and 42 days after immunization (Figure 3). The functional activities of the sera from the vaccinated mice were investigated by comparing the antibody titers against lysates from cells infected with the MCMV Smith strain with those against lysates from cells infected with the MCMV mutant m-M78, which was derived from the MCMV Smith strain and contained a deletion of the M78 open reading frame. MCMV mutants inactivating M78 replicated efficiently in NIH 3T3 cells in vitro but were attenuated in growth in mice [24,25].

Sera obtained from mice immunized with v-M78 exhibited at least 250 times higher antibody titers against MCMV Smith-infected cell lysates than those with the control vaccine v-C (Figure 3A). However, the sera from both mice immunized with v-C and v-M78 displayed low antibody titers against the m-M78-infected cell lysates (Figure 3B). These results suggest that the IgG humoral responses elicited by v-M78 were specifically against M78.

To investigate the mucosal antibody responses elicited by the vaccines, we also examined the anti-MCMV IgA levels in the nasal wash at 0, 16, 32, and 42 days after immunization. At 42 days post immunization, mice immunized with the vaccine v-M78 displayed more than 60 times higher IgA levels against Smith-infected cell lysates compared to those immunized with the control v-C (Figure 3C). In contrast, the mucosal washes from both mice immunized with v-C and v-M78 displayed low IgA antibody titers against the m-M78-infected cell lysates (Figure 3D). These results suggest that v-M78 elicited both M78-specific serum IgG and mucosal IgA responses.

### 3.4. T Cell Responses Elicited by Salmonella-Based Vaccines

**Figure 4:**
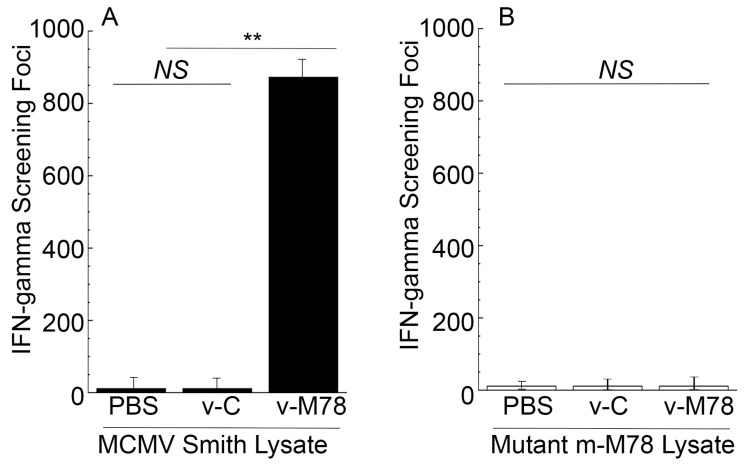
T cell responses in mice treated with PBS, the control vaccine v-C, and the functional vaccine v-M78. We treated animals intragastrically with PBS only, v-C, and v-M78 at days 0, 14, and 28. Splenocytes (n = 5) were isolated at 42 days post immunization and stimulated with Smith-infected (**A**) and m-M78-infected cellular lysates (**B**) for 48 h. We assayed the T cell responses with an ELISPOT analysis of the IFN-γ production by the cells. The results were expressed as spot-forming cells (SFCs) per million cells. ** *p* < 0.05. NS, not significant. Error bars indicate the standard deviation. Two trials were conducted with a total of 60 animals, and each trial included 30 animals (10 mice per group). Experiments were conducted in duplicate and repeated three times.

We harvested splenocytes (n = 1 × 10^6^ cells) from animals 42 days after oral administration and stimulated them with the lysates of cells infected with the Smith strain or m-M78 mutant. The functional vaccine v-M78 induced about 100 times higher anti-MCMV IFN-γ-producing T cell responses against the Smith-infected cell lysates than the control vaccine v-C (Figure 4A). In contrast, mice immunized with v-C and v-M78 displayed low anti-MCMV T cell responses against the m-M78-infected cell lysates (Figure 4B). Thus, v-M78 appeared to elicit M78-specific T cell responses.

### 3.5. Salmonella Vaccine-Elicited Immune Protection of Mice from Intraperitoneal MCMV Challenge

**Figure 5:**
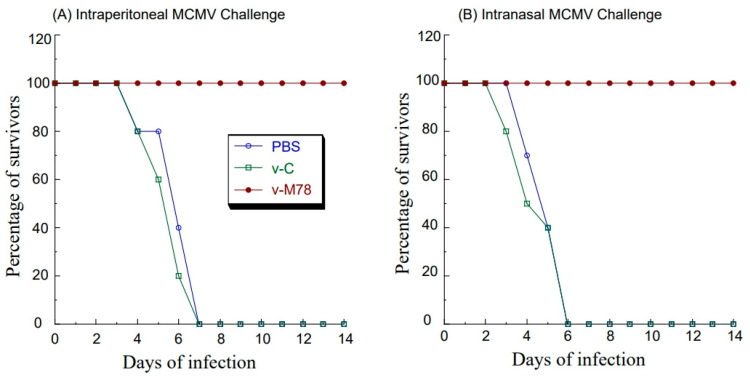
Immune protection of mice from MCMV challenge. Groups of mice (10 mice per group) were intragastrically immunized three times at days 0, 14, and 28 with PBS only, control vaccine v-C (v-C), and functional vaccine (v-M78) and then challenged intraperitoneally (**A**) or intranasally (**B**) with salivary gland-passaged MCMV Smith (1 × 10^6^ PFU) at 42 days post initial immunization. Mice were monitored for survival, and results are presented in terms of percent survival.

To study if v-M78 elicited immune protection against a systemic MCMV challenge, mice were vaccinated with v-M78 and v-C at days 0, 14, and 28 and then intraperitoneally challenged with a lethal dose of salivary gland-passaged highly pathogenic MCMV at day 42 (i.e., two weeks after the final immunization). Mice vaccinated with the functional vaccine v-M78 showed 100% protection against the MCMV challenge after 14 days post challenge, while mice administered with the control vaccine v-C or PBS exhibited no protection against the MCMV challenge as all these animals died within 7 days post challenge (Figure 5A).

**Figure 6:**
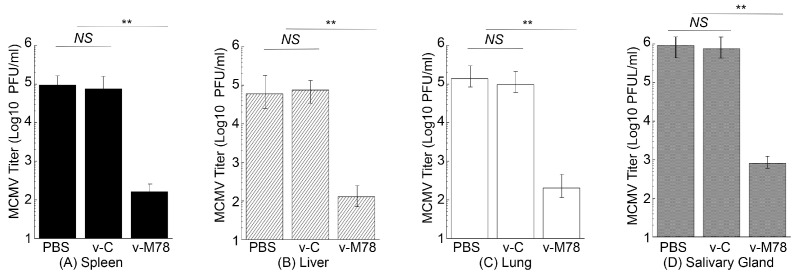
Virus titers in the spleens (**A**), livers (**B**), lungs (**C**), and salivary glands (**D**) of mice after the intraperitoneal MCMV challenge. We treated mice intragastrically with PBS, v-C, and v-M78 at days 0, 14, and 28 and then challenged them intraperitoneally with MCMV Smith (5 × 10^4^ PFU) two weeks after the final immunization. Viral titers were assayed from organs collected 5 days post challenge. The limit of detection was 10 PFU/mL. ** *p* < 0.05. NS, not significant. Error bars indicate the standard deviation. Two trials were conducted with a total of 60 animals, and each trial included 30 animals (10 mice per group). Experiments were conducted in duplicate and repeated three times.

To further investigate the immune protection elicited by v-M78, we measured virus loads in different organs of the immunized mice after the MCMV challenge. Mice were vaccinated with v-M78 and v-C at days 0, 14, and 28 and then intraperitoneally challenged with sub-lethal MCMV doses at day 42. Mouse organs were harvested at day 5 post challenge, and viral titers in these organs were quantified. The virus titers in the spleens, livers, lungs, and salivary glands in v-M78-vaccinated mice were about 600-, 500-, 700-, and 1000-fold lower than those in PBS-treated mice, respectively (Figure 6). In contrast, virus titers in these organs from animals vaccinated with the control vaccine v-C exhibited no significant difference compared to those from the PBS-treated animals (Figure 6A–D). These results suggest that the functional vaccine v-M78 provides immune protection against MCMV and reduces the infection from and replication of the challenged MCMV in mice.

### 3.6. Salmonella Vaccine-Elicited Immune Protection of Mice from Intranasal MCMV Challenge

Attenuated *Salmonella* strains can induce strong mucosal immune responses due to their infection and colonization in the gut. Our results also indicated the v-M78-mediated induction of anti-MCMV IgA in the mucosal nasal wash (Figure 3C,D). Thus, it is reasonable to suggest that intragastrical vaccination with v-M78 may elicit mucosal immune responses against an intranasal MCMV challenge. To determine if this was the case, two series of experiments were carried out. In the first series of experiments, mice were vaccinated with v-M78 and v-C at days 0, 14, and 28, then intranasally challenged with lethal doses of salivary gland-passaged highly pathogenic MCMV at day 42, and finally closely monitored for survival for 14 days post challenge. All mice administered with v-C or PBS exhibited no protection against the MCMV challenge and died within 6 days post challenge (Figure 5B). In contrast, 90% of the mice vaccinated with v-M78 remained alive and healthy after 14 days post challenge (Figure 5B).

**Figure 7:**
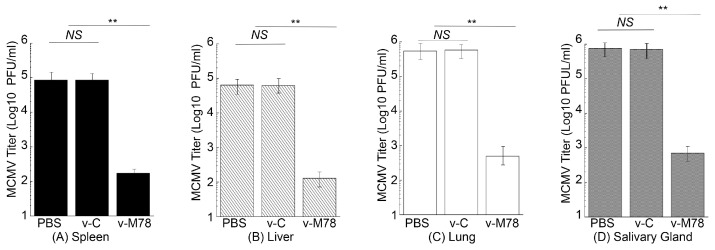
Virus titers in the spleens (**A**), livers (**B**), lungs (**C**), and salivary glands (**D**) of mice after the intranasal MCMV challenge. We treated mice intragastrically with PBS, v-C, and v-M78 at days 0, 14, and 28 and then challenged them intranasally with MCMV Smith (5 × 10^4^ PFU) two weeks after the final immunization. Viral titers were assayed from organs collected 5 days post challenge. The limit of detection was 10 PFU/mL. ** *p* < 0.05. NS, not significant. Error bars indicate the standard deviation. Two trials were conducted with a total of 60 animals, and each trial included 30 animals (10 mice per group). Experiments were conducted in duplicate and repeated three times.

The second series of experiments was to measure virus loads in different organs of the immunized mice after the intranasal MCMV challenge. Mice were vaccinated with v-M78 and v-C at days 0, 14, and 28 and then intranasally challenged with sub-lethal MCMV doses at day 42. At day 5 post challenge, organs were collected and viral titers were quantified. The virus titers in the spleens, livers, lungs, and salivary glands in v-M78-vaccinated mice were about 500-, 500-, 1000-, and 1000-fold lower than those in PBS-treated mice, respectively (Figure 7). In contrast, virus titers in these organs from animals vaccinated with the control vaccine v-C exhibited no significant difference compared to those from the PBS-treated animals (Figure 7A–D). These results suggest that the functional vaccine v-M78 induces mucosal immune protection against intranasal MCMV infection.

## 4. Discussion

Human CMV continues to pose a significant public health concern as the leading viral cause of congenital infections resulting in intellectual disability and hearing loss [1,5,6]. There is currently an urgent need to develop effective vaccines against HCMV infection and its associated human diseases. Oral anti-CMV vaccines may represent attractive candidates because they are cost-effective and administered easily. In the current report, we described how we generated a new attenuated *S. typhimurium* strain, SL416, that acts as a gene transfer vector to express the MCMV M78 protein. The *Salmonella*-based vaccine v-M78 induced anti-MCMV humoral and T cell responses and elicited effective immune protection against an MCMV challenge in mice. These results provide the first direct evidence that a *Salmonella* (ssrA/B^−^)-based vaccine, v-M78, is effective against MCMV infection in mice.

Attenuated *Salmonella* represent unique and promising oral vaccine vectors against pathogens [16]. For example, the vaccine against typhoid fever is derived from an attenuated *Salmonella* strain [42,43]. However, *Salmonella*-based oral vaccines have not been reported against CMV infection and its associated diseases. In our study, the attenuated *Salmonella* strain SL416 was derived from the auxotrophic strain SL7207 [33] and contained a deletion of part of ssrA/B. SsrA/B is required for the expression of many *Salmonella* pathogenicity island 2 (SPI-2) genes [22]. Thus, SL416, with a deletion in ssrA/B, should have little capability to kill mice and cause substantial pathological effects in vivo (Figure 1). Moreover, SL416 exhibited excellent gene transfer activity. The M78 protein was detected in cells and in mice treated with v-M78 (Figure 2). In mice, v-M78 induced strong anti-MCMV humoral and T cell immune responses and elicited effective immune protection against MCMV infection (Figure 3, Figure 4, Figure 5, Figure 6 and Figure 7). Therefore, strain SL416 represents a new and promising oral vaccine vector against CMV infection.

One of the most important issues related to the use of live bacteria as a vaccine is the safety of these vaccines and their potential pathogenesis in vaccinated individuals. Previous studies showed that a vaccine against typhoid fever was derived from an attenuated *Salmonella* strain [42,43], demonstrating that attenuated *Salmonella* is safe for the vaccination of humans. Another issue is related to the turnover/clearance of the bacteria cells post vaccination. The construction of bacterial mutants with the inactivation of virulence factors important for virulence/pathogenesis and immune evasion should reduce their ability to cause pathological effects and increase their clearance/turnover by the immune system. Little is currently known about the turnover/clearance of our constructed vaccines, v-C and v-M78, in the vaccinated mice. Different approaches, such as qPCR to measure the bacterial DNA and numerous imaging methods to visualize the bacteria, can be performed to study this issue. In our experiments, a high dose of *Salmonella* was introduced to the animals, yielding a satisfactory protection and survival rate. It is important to use fewer bacteria to minimize potential safety issues associated with live *Salmonella*. Further studies can be carried out to address this issue with the improved design and construction of *Salmonella* strains exhibiting higher immunogenicity and expressing higher levels of the antigen. These studies will facilitate the development of attenuated *Salmonella* as vaccine vectors for clinical applications.

It is currently not completely understood how attenuated *Salmonella* strains carry out gene transfer in infected mammalian cells. In the delivery system reported here, we constructed the attenuated *Salmonella* strain SL416 and generated v-M78 by introducing peM78 into SL418. In *Salmonella*-infected cells, the bacteria release transgene expression constructs (i.e., peM78), which enter the nuclei, resulting in transgene expression [17,18]. Additional studies on understanding SL416-induced gene transfer for M78 expression will facilitate the development of better *Salmonella*-based vectors for the construction of oral vaccines against human diseases.

Vaccines being explored against CMV include conventional inactivated whole-viral antigen vaccines, recombinant protein vaccines, virus-like particle (VLP) vaccines, DNA vaccines, and vaccines based on live attenuated virus vectors [7]. However, there are currently no FDA-approved anti-CMV vaccines available. The MCMV infection of mice represents an excellent model for studies of the immune responses to CMV infection and anti-CMV vaccine development. For example, DNA-based and viral vector-based vaccines expressing different MCMV antigens elicited strong antiviral humoral and T cell responses and inhibited viral infection and protected mice from a MCMV challenge [9,10,11,12,13]. However, it has not been reported whether M78 or its HCMV homolog, UL78, can serve as an antigen for vaccine development. Previous studies have implicated the important roles of the M78 and UL78 proteins in viral infection and pathogenesis. The M78 and UL78 proteins function as G protein-coupled receptor (GCR) homologs and are expressed in both virions and infected cells [23]. M78 regulates viral IE mRNA expression during viral replication and down-regulates MHC II expression during viral salivary gland colonization [24,25]. Because they are virion components, M78 and UL78 are considered potential vaccine candidates against CMV infections. However, whether M78 or UL78 functions as an anti-CMV immunogen has not been reported. Our results imply that M78 and UL78 may be promising antigens for anti-CMV vaccine development. Further studies on these issues will facilitate the development of novel vaccines against CMV infections and their associated diseases.

It should be noted that the immune responses observed in a mouse model may not reflect those in humans [3,8]. We need to carry out additional investigations to understand the immune responses to *Salmonella*-based CMV vaccines in humans. These studies will provide insight into our understanding of the use of *Salmonella*-based vaccines against CMV and associated diseases.

## 5. Conclusions

Human CMV is the most common cause of viral congenital infections worldwide. The development of effective vaccines against human CMV infection and disease is a high priority. In this study, an attenuated *Salmonella* strain was generated as an oral vaccine vector for the delivery and expression of the M78 protein of murine cytomegalovirus (MCMV). Using the MCMV infection of mice as the CMV infection model, we characterized the immune responses and protection induced by the constructed *Salmonella*-based vaccine. Our results provide the first direct evidence that a *Salmonella*-based vaccine expressing M78 elicits strong humoral and cellular immune responses and induces immune protection against MCMV infection. Furthermore, our study demonstrates the potential of using *Salmonella*-based oral vaccines against CMV infection.

## Data Availability

The dataset is available on request from the authors.
